# Manganese-induced cellular disturbance in the baker’s yeast, *Saccharomyces cerevisiae* with putative implications in neuronal dysfunction

**DOI:** 10.1038/s41598-019-42907-2

**Published:** 2019-04-25

**Authors:** Raúl Bonne Hernández, Houman Moteshareie, Daniel Burnside, Bruce McKay, Ashkan Golshani

**Affiliations:** 1 University Federal de São Paulo Departamento de Química, Laboratorio de Bioinorgânica e Toxicologia Ambiental – LABITA, Rua Prof. Artur Riedel, 275, CEP: 09972-270, Diadema, SP Brazil; 2 Department of Biology and Ottawa Institute of Systems Biology, Carleton University, 1125 Colonel by Drive, Ottawa, ON K1S 5B6 Canada

**Keywords:** Bioinorganic chemistry, Neurodegeneration

## Abstract

Manganese (Mn) is an essential element, but in humans, chronic and/or acute exposure to this metal can lead to neurotoxicity and neurodegenerative disorders including Parkinsonism and Parkinson’s Disease by unclear mechanisms. To better understand the effects that exposure to Mn^2+^ exert on eukaryotic cell biology, we exposed a non-essential deletion library of the yeast *Saccharomyces cerevisiae* to a sub-inhibitory concentration of Mn^2+^ followed by targeted functional analyses of the positive hits. This screen produced a set of 43 sensitive deletion mutants that were enriched for genes associated with protein biosynthesis. Our follow-up investigations demonstrated that Mn reduced total rRNA levels in a dose-dependent manner and decreased expression of a β-galactosidase reporter gene. This was subsequently supported by analysis of ribosome profiles that suggested Mn-induced toxicity was associated with a reduction in formation of active ribosomes on the mRNAs. Altogether, these findings contribute to the current understanding of the mechanism of Mn-triggered cytotoxicity. Lastly, using the Comparative Toxicogenomic Database, we revealed that Mn shared certain similarities in toxicological mechanisms with neurodegenerative disorders including amyotrophic lateral sclerosis, Alzheimer’s, Parkinson’s and Huntington’s diseases.

## Introduction

All trace elements play an important role in the balance of life on our planet. The ability of cells to effectively utilize these elements depends strongly on their concentration, chemical speciation and fractionation^1^. Manganese (Mn) is the twelfth most abundant element in the earth’s crust. Natural levels of Mn range from 1–200 μg/L in fresh water and 410–6700 mg/kg (dry weight) in sediment^2^. In aquatic environments, Mn^2+^ is the most dominant and stable water-soluble species when the pH and redox potential are kept low^2^. The Mn^3+^ ion is soluble only in complex form and Mn^4+^ has very limited solubility^3^. Different Mn species can interconvert via oxidative or reductive processes depending on the redox environment^1,3^. Additionally, it is known that Mn is an vital trace mineral in nutrition^4^. Locally, levels of Mn can rise significantly in certain areas due to geogenic factors or anthropogenic activities such as mining^5–8^. Higher levels of Mn can have negative consequences for environmental health.

Epidemiological and toxicological studies suggest that Mn can be detrimental to specific biological processes and beneficial to the others in a concentration-dependent manner. This is also influenced by developmental stage, disease state^9,10^ cell type^11^, and/or the organism itself. Cell models from various organs including the liver, kidney and brain, suggest that neurotoxicity is the principal effect of this metal^11^. However, the underlying mechanism(s) are unclear and remain the subject of current studies^12^. Some investigations provide strong evidence that Mn can disturb the vital flow of genetic information from DNA to RNA to protein^13–16^. Recently; an interesting *in vitro* study demonstrated that Mn^2+^ had similar effects that Fe^2+^ and Mg^2+^ on rRNA folding and it can replace Mg^2+^ as the dominant divalent cation during translation of mRNA to functional protein^17^.

The addition of MnCI_2_ (5 mM) to highly-purified membrane rat-liver fractions caused a 30% increase in the polysome-binding capacity of stripped rough endoplasmic reticulum (ER) membranes, while four- to five-fold increases were observed with smooth ER membranes^15^. Previous studies in yeast suggest that the Mn^2+^ inhibits protein synthesis, disrupts nuclear DNA replication, and demonstrates mutagenic activity when under selective pressure^13,14^. Overwhelming evidence indicates that when stressed or undergoing environmental adaptation, cells accumulate non-synonymous mutations^18^. Studies investigating Mn-trafficking in humans suggest that Mn-induced Parkinsonism can result from mutations in *SLC30A10*, *ATP13A2* or *ZnT10*^19,20^. Also, a His → Asn reversion mutant in *ZnT10* conferred Mn transport activity and loss of zinc transport activity^20^. *ZnT10* codes for a protein that is localized to the plasma membrane and is involved in zinc subcellular homeostasis^20^, while *SLC30A10* codes for a surface-localized Mn efflux transporter that reduces cellular intake of Mn and protects against Mn-induced toxicity in neurons and worms^19^. The synthesis of these proteins is controlled by ribosomal activity in connection with the ER^21^. The ER is a large, continuous membrane-bound organelle with distinct domains and numerous contact sites with the plasma membrane, Golgi, mitochondria, and other cellular components including the SNARE (soluble N-ethylmaleimide-sensitive fusion protein attachment protein receptors) complex that participates in the ER formation, fusion and function^22^. Protein synthesis is a crucial process for all living cells. Due to its central importance to cell survival and high energy requirements, protein synthesis is firmly regulated and strongly connected to other cellular processes, including the cell cycle and metabolic pathways^23,24^. Also, the mechanisms that govern the protein synthesis are highly-conserved through the course of evolution from higher to lower eukaryotes, as well as prokaryotes. In line with this, several aggregation-prone yeast proteins have human homologues that are implicated in protein misfolding associated diseases, suggesting that similar mechanisms may apply in both organisms and that yeast can serve as a good model organism to study such processes^25^. Studies using ribosome profiling and/or polysome profiling and classic gene expression analyses have provided new insights into the identification of novel genes that can affect this process as well as the mechanism of protein synthesis itself, which is often considered the endpoint of gene expression^26,27^.

Compound genome-wide toxicity is best studied using a systems biology approach, which can decipher the role(s) of individual components of complex biological systems under certain conditions by examining interactions on a global scale. To this end, large-scale chemical-genomic studies using yeast have been employed to identify individual chemical-genetic interactions (CGIs) and generate interaction profiles to infer mechanism(s) of action^28,29^. A category of these interactions occurs when the deletion of a single gene causes significant sensitivity or resistance to a target compound. Such interactions can suggest a functional relationship between the deleted gene and cell’s responses to the target compound. By screening for chemical-genetic interactions across the genome, significant insights into genotoxicity pathways can be drawn. In a similar context, protein-protein interaction (PPI) networks also provide a useful resource to better understand the mecheanism of toxicity. PPIs underlay nearly all biological processes, including cell-to-cell interactions, and metabolic and developmental controls. Depending on structural properties and functional characteristics, PPIs can range from transient interactions that generally participate in signalling pathways tothemore permanent interactions required to form stable protein complexes. It has been revealed that over 80% of proteins do not operate alone but in complexes^30,31^. PPIs can be studied in vitro, in vivo, and in silico^31^.

Connections between environmental toxin exposure and several human diseases have stimulated increased investigation into the toxicity of environmental contaminants using different model organisms^32,33^. Despite the evolutionary distance between yeast and humans, the underlying molecular players of numerous important pathways including programmed cell death, cell cycle progression and gene expression are conserved between the two species, allowing for the study of neurotoxins using highly-developed omics approaches in yeast^34^. Mn is one such compound that is applicable for large-scale screening in yeast. However, the range of intra-cellular Mn concentrations with physiological relevance or toxicity is quite large. Particularly, various studies done in yeast suggest that concentrations can range from between 2–100 nmol of Mn/(10 × 10^9^ cells), or 0.04–2.0 mM Mn (assuming a single yeast cell has a volume of 50 femtoliters), without any impact on cell growth^35^. However, at levels below or above this, Mn induces toxicity stimulating cellular responses, including upregulating or downregulating cell surface and intra-cellular transport systems^35^. Consequently, yeast has been used to study events associated with Mn homeostasis, neurotoxic cell death, and neurodegeneration^34–37^. Generally, these studies have used Mn concentrations above of 2 mM, but have hardly explored the large network of physiological pathways that involve Mn, and hence the role of Mn in these processes remains unclear. In the current study, we provide evidence to connect Mn toxicity to the gene expression pathway in yeast. The impairment of protein biosynthesis by Mn^2+^ revealed in this study improves our current understanding of Mn-induced neurotoxicity and neurodegenerative disorders such as including amyotrophic lateral sclerosis (ALS), Alzheimer’s disease (AD), Parkinson’s disease (PD) and Huntington’s disease (HD).

## Results

**Figure 1:**
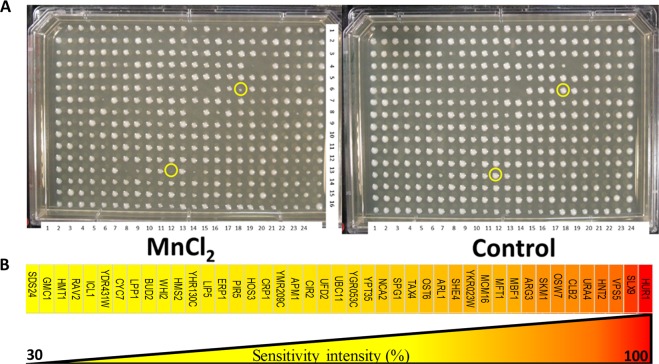
Representative illustration of Mn-induced disruption in yeast gene deletion array after exposure to MnCl_2_ (1.35 mM) for 24 hours. Mutants that showed a relative reduction in growth (sensitivity) of 30% or more were selected as hits (p < 0.05). Examples of hit stains are indicated using yellow circles.

To identify pathways that are influenced by Mn exposure, we screened for gene deletion strains that demonstrate increased sensitivity to Mn^2+^ using the yeast non-essential gene deletion array (yGDA), Fig. 1A. These types of screens can provide a CGI profile for a target toxin and contribute to our knowledge of the cell’s global stress responses to that toxin. To this end, we performed sensitivity analysis by screening approximately 4700 gene deletion strains, under two conditions (presence and absence of MnCl_2_), for a total of approximately 28,000 individual analyses. Sensitivity was investigated by determining the relative colony growth size in the presence/absence of the target compound. In this way, we identified 68 gene deletion mutants with significantly altered growth profiles (Supplementary Material, Table SM1), of which 43 were confirmed to display high sensitivity to a sub-inhibitory concentration (1.35 mM) of Mn (a high concentration of a bioactive/toxic compound where growth of a wildtype (WT) strain is not completely inhibited), Fig. 1B. These genes represent a CGI profile for Mn sensitivity. They often represent “double hits” where the gene deletion and Mn treatment target compensating pathways generating an aggravated effect. The hits identified here were then subjected to further analysis.

### Functional proteomic and Gene Ontology (GO) analysis of sensitive mutants identifies multiple pathways including protein synthesis

**Figure 2:**
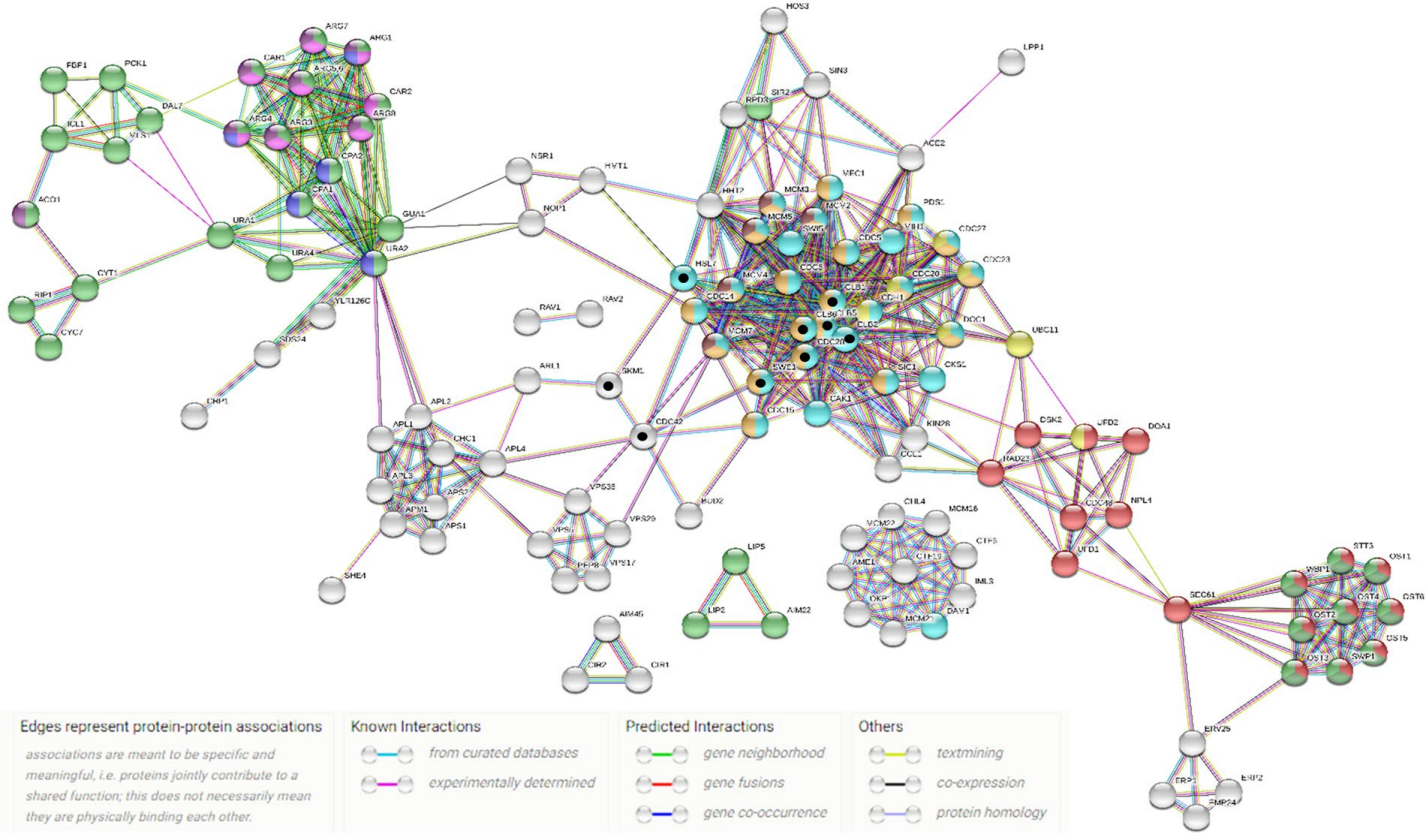
The inferred and enriched PPI network from 43 genes that sensitize yeast to Mn when deleted. Analysis performed using the String database. Network properties are as follows: The minimum required interaction score, to be included at the predicted network, was accepted with a threshold on the high confidence equal 0.7; Number of nodes: 143; Number of edges: 594; Expected number of edges: 287; Average (avg) node degree: 8.31; avg. local clustering coefficient: 0.652; PPI enrichment p-value: <1.0e-16. Nodes and edges represent proteins and PPIs, respectively. Red nodes (protein processing in endoplasmic reticulum); blue nodes (metabolic pathways); dark green nodes (N-Glycan biosynthesis); cyan nodes (cell cycle); yellow nodes (ubiquitin-mediated proteolysis); orange nodes (meiosis); maroon nodes (DNA replication); purple nodes (amino acid biosynthesis); magenta nodes (arginine and proline metabolism); lime green nodes (alanine, aspartate and glutamate metabolism). The protein with black points in the center represent the MAPK signaling pathway. Proteins that are not connected to at least one partner are not shown.

To have a comprehensive coverage of the hits identified in our sensitivity screen, the String database was used to expand the obtained CGI profile for Mn on the basis of PPI data^38^. String uses physical interactions and functional associations to study a defined set of proteins and expand it by including associated proteins. In this way, the network of functional interactors for Mn was increased to approximately 600 edges (p-value < 1.0e-16), of which more than 85% are known interactions. For example, approximately 87% of the interactions have been experimentally verified and almost 98% are from curated databases. A schematic representation of these interactors is shown in Fig. 2.

Enrichment of cellular pathways represented by the expanded list of proteins is shown in Table 1. As expected, proteins associated with cellular development and protein metabolism were highly enriched^39^. Particularly, the ER associated activities have been highly connected to Mn toxicity^21,40–43^. However, a direct connection between protein synthesis and Mn toxicity has not been previously reported. This led us to further investigate the influence of Mn toxicity on protein biosynthesis.

**Table 1:** Cellular pathways enriched in the Mn-induced interaction network.

Cellular Processes	Pathway description	Number of observed genes	FDR
Cell cycle	Cell cycle	29	8.46E-21
	Meiosis	21	1.26E-11
	DNA replication	5	4.39E-03
Biosynthesis and Metabolism of Proteins	Protein processing in endoplasmic reticulum	17	1.78E-11
	N-Glycan biosynthesis	9	2.09E-07
	Arginine and proline metabolism	8	2.84E-06
	Ubiquitin mediated proteolysis	7	6.42E-04
	Alanine aspartate and glutamate metabolism	5	4.12E-03
	Biosynthesis of amino acids	8	3.47E-02
Metabolism	Metabolic pathways	36	2.83E-06

This table was produced by performing GO analysis on the PPI network associated with genes influenced by Mn exposure (Fig. 1). This network was generated using the String database.

**Figure 3:**
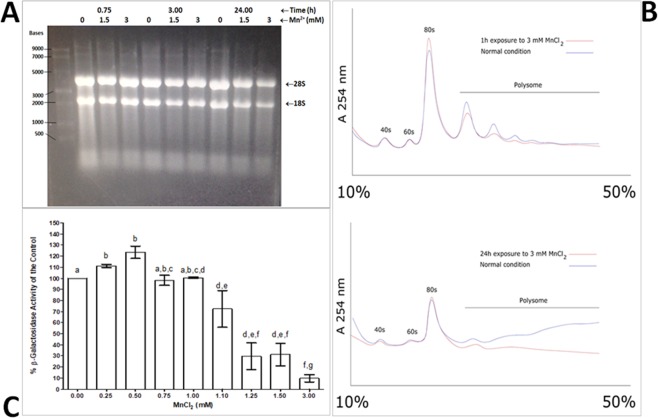
Influence of Mn on protein biosynthesis. (**A**) rRNA levels are reduced in response to Mn (1.5 and 3 mM), for 0.75, 3 and 24 hours. Total rRNA (1 µg) run on a 1.2% agarose gel. (**B**) Ribosome profile analysis suggests that the number of active polysomes are reduced in response to Mn 3 mM, for 1 and 24 hours. (**C**) The relative expression of β-galactosidase is reduced in response to the presence on increasing concentrations of Mn. Bars represent the mean value of at least 3 independent experiments and error bars represent (mean ± SEM). Differences were stipulated by ANOVA one-way, followed by a Bonferroni post-test. Letters indicate statistically significant differences among treatments (p < 0.05).

To study if Mn may also affect gene expression at the translation level, total RNA levels were analyzed. In response to the presence of Mn, we observed decreased levels of rRNA molecules (Fig. 3A). After treating the cells with 1.5 mM and 3.0 mM Mn for 45 minutes, total rRNA levels are reduced in a dose-dependent manner. We repeated this experiment by increasing the duration of Mn treatment to 3 and 24 hours. We observed similar results indicating that total levels of rRNA molecules seem to be reduced in response to Mn. Next, we investigated ribosome profiles of cells in response to Mn treatment (Fig. 3B). We observed a reduction in the pool of polysomes in response to treatment with 3 mM Mn for 1 hour, in addition to an increase in the pool of 80S ribosomes. Reduction in polysomes is interpreted as a decrease in the number of ribosomes that are active and engaged in synthesizing proteins. An increase in 80S monosomes is generally regarded as stalled initiation of translation. Treatment of the cells with Mn for 24 hours, resulted in additional reduction in polysomes in comparison to control conditions.

Lastly, using an expression vector we investigated the expression of *β-galactosidase*, used as a reporter, in response to Mn. In a dose-dependent manner, the presence of Mn^2+^ reduced the expression of β-galactosidase (Fig. 3C). Importantly, this trend differs significantly when other divalent ions such as Ca^2+^, Mg^2+^ and Zn^2+^ are used suggesting that the decreased rate of translation is unique to Mn^2+^ stress and not a general byproduct (Supplementary Material, Fig. SM2). Altogether, these follow-up investigations connect Mn toxicity to the process of protein biosynthesis, which were identified as an enriched cellular process in our GDA analysis.

### Manganese-induced disturbance of processes that converge to protein biosynthesis in yeast, which mimics molecular pathways associated with neurodegeneration

It has been postulated that the ER has various active domains and membrane contact sites that are required for multiple cellular processes including protein and lipid biosynthesis, calcium regulation, and the exchange of macromolecules^22^. In this study, multiple approaches including GDA and PPI analysis (Figs 1 and 2), GO ontology enrichment (Table 1), total rRNA analysis, ribosome profiling and a β-galactosidase reporter assay (Fig. 3), together suggest that the Mn induces a significant perturbation of protein biosynthesis and associated pathways.

**Figure 4:**
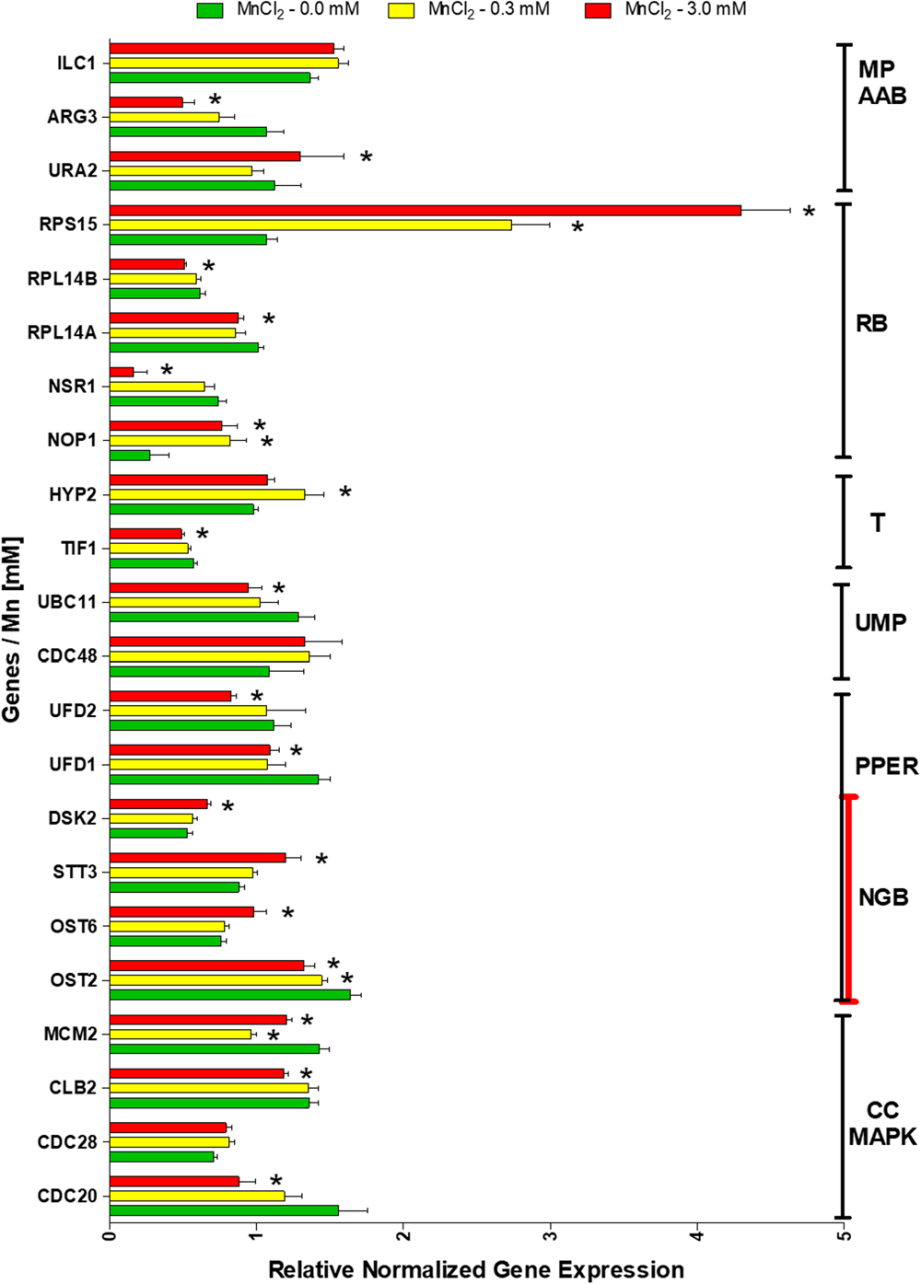
Relative gene-transcription of preselected hits from yeast stressed with Mn, after analysis of protein-protein interaction network (figure 2), β-galactosidase expression assay and ribosome profile, which are an evidence of perturbation of protein biosynthesis and other associated pathways (protein processing in endoplasmic reticulum, metabolic pathways, N-Glycan biosynthesis, cell cycle, ubiquitin mediated proteolysis, amino acid biosynthesis, MAPK signaling pathway and translation control analysis) was performed by qPCR. Bars represent the mean value of at least 3 independent experiments and error bars represent (mean ± SEM). Preliminarily, we verified some trends to be different between Mn treatment and the control using t-test (^+^p < 0.05). Then, we confirmed several significant differences by ANOVA two-way, followed of Bonferroni post-test (*p < 0.05).

To augment this finding and more-closely study individual participants, we selected several genes linked to processes that converge on protein biosynthesis and then analyzed their relative transcription levels using qPCR (Fig. 4). Indeed, the presence of Mn induced alterations in the expression of these genes. For example, we observed decreased expressionof key translation initiation factor eIF4A (*TIF1)* and upregulation of the essential translation elongation factor eIF-5A (*HYP2*). Additionally, several other genes associated with translation and/or ribosome biogenesis had significantly altered levels of transcription including the downregulation of *NSR1*, *NOP1* and up-regulation of the gene *RPS15*. However, protein biosynthesis is a complex process that involve other pathways. For instance, we observed perturbation in the expression of genes such as *UFD1*, *UFD2*, *STT3*, *DSK2*. Additionally, *OST2* and *OST6*, involved in post-translational processing in the ER (PPER), were significantly downregulated and upregulated, respectively. Similar alterations are inferred for the N-glycan biosynthesis pathway, which is partially regulated by *OST2* and *OST6* activity. We also observed decreased-expression of *ARG3* involved in amino acid biosynthesis, including arginine, proline, alanine, aspartate and glutamate metabolism and increased expression of *URA2*.

Also of interest, genes such as *CDC20* and *UBC11*, which are related to ubiquitin proteolysis were significantly disrupted. Protein synthesis underpins much of cell growth and multiplication^44^. Coincidently, impairment of *CDC20* suggests a direct relationship among dysregulation of protein biosynthesis and alteration of MAPK signaling pathways, cell cycle and DNA replication respectively.

### Mn-induced molecular impairment in yeast mimics pathways associated with neurodegeneration

**Figure 5:**
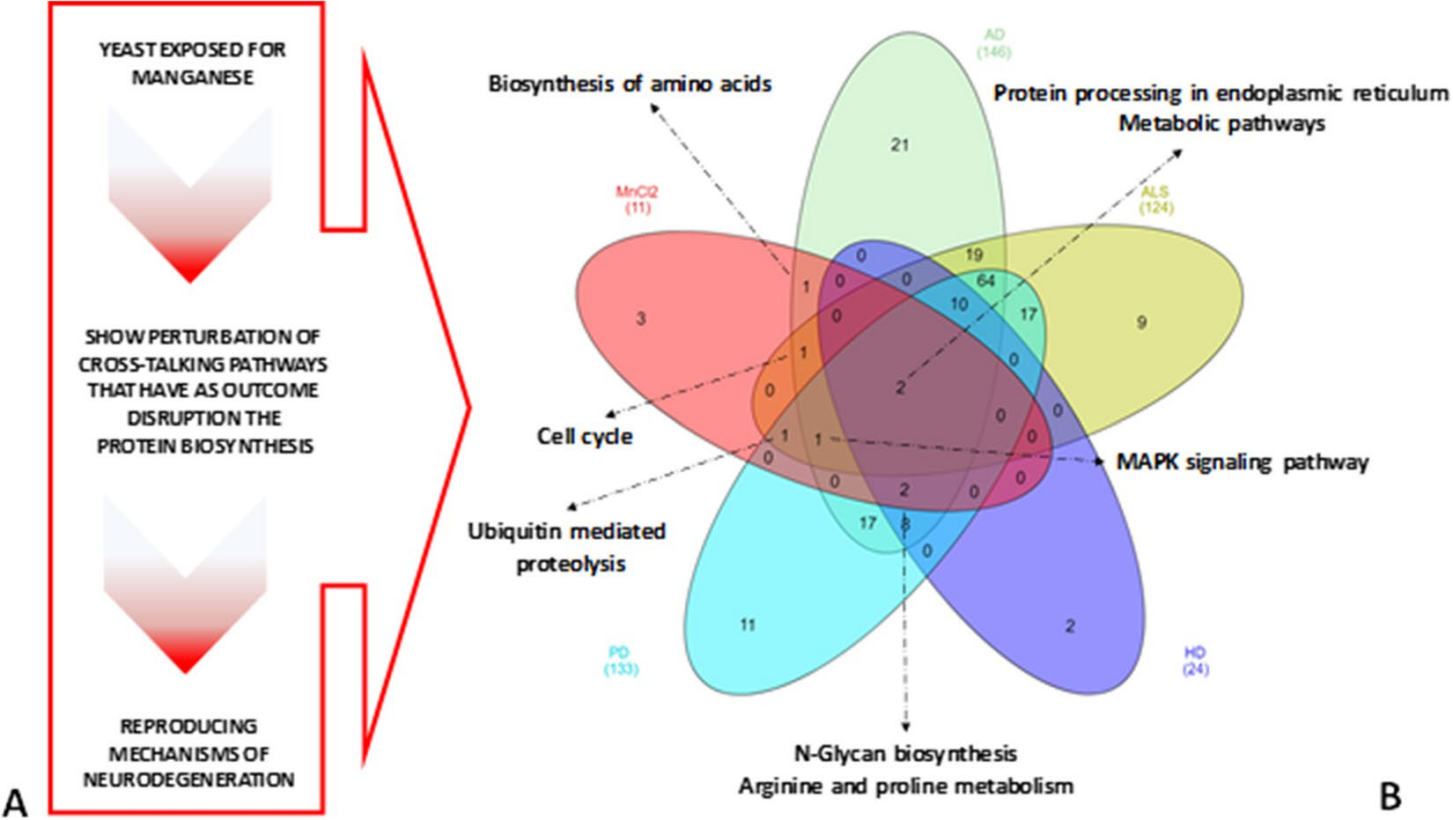
Mn-induced disruption pathways in yeast (**A**) share similarities with certain pathways linked to neurotoxicity and neurodegeneration (**B**). Analysis performed with the Comparative Toxicogenomic Databasewhich containscurated data regarding the mechanisms of action for neurodegenetative disorders. The results these analyses indicate overlapping pathways related to Mn Toxicity, AD (Alzheimer’s Disease), Amyotrophic Lateral Sclerosis (ALS), HD (Huntington’s Disease), and PD (Parkinson’s Disease).

We identified alterations in various pathways that lead to impairment of protein biosynthesis, which is a conflicting topic in neurodegeneration research. Some reports have viewed this as a therapeutic target, while others suggest that it provokes the onset of certain neurodegenerative disorders^45^. Due to the conservation of the key cellular processes and genes, yeast has been used as a model organism to study human neurodegenative diseases^34^. In this sense, we conducted an additional analysis of the pathways affected by Mn using both the String database and the Comparative Toxicogenomics Database – CTD^46^, which permits the development of novel hypotheses about the relationships between chemicals and diseases^47^. The results are shown in Fig. 5.

We verified that approximately 31% (44 proteins/genes, Supplementary Material - Fig. SM 3) of the inferred network for hits (genes) affected by Mn (Fig. 2) have homologues in human, of which approximately 73% (32 proteins/genes, Supplementary Material – Fig. SM 3) are potentially linked to neurodegeneration, according to the CTD^46^. The genes affected by Mn suggest that this cation-induced toxicity in yeast involves disruption of several pathways which together lead to impairment of protein biosynthesis (Fig. 5A). These alterations shared characteristics with pathways involved in neurodegenerative diseases (Fig. 5B). For example, we identified that the MnCl_2_ affects the *CDC20* involved in the metabolism of proteins^46^,the cell cycle and MAPK signaling pathways and is potentially involved in the development of neurodegenerative disorders such as AD, ALS, HD and PD^46^. At the same time, *UFD1* is associated with protein processing in endoplasmic reticulum and potentially linked to the evolution of AD, ALS and PD^46^. Altogether; our findings and subsequent inferences suggest that the developmental impairment induced by Mn, according to cell cycle disruption, is mainly influenced by collective perturbation of pathways that converge to disturbance of protein biosynthesis. We demonstrated a decrease in total RNA, polysome and β-galactosidase activity as well as potential alterations in the expression of genes directly associated with translation (*HPY2* and *TIF1)* and ribosome biogeneses (*RPS15*, *NSR1* and *NOP1*). Together findings suggest a plausible hypothesis for Mn-induced neurotoxicity and neurodegeneration (Fig. 5B). Further analysis in higher-order animal models is needed to confirm this theory.

## Discussion

The role of Mn in toxicity, particularly in relation to neurotoxicity and neurodegeneration disorders, remains unclear with several proposed hypotheses^12^. The CTDdescribes Mn as an essential trace element, with possible connections to approximately 570 biological processes and/or pathways^48^. In this work, using a functional genomics and systems biology approach, we observe a connection between Mn and cellular processes such as cell cycle progression, cell signaling, and protein metabolism. Agreeably, previous studies have suggested the possibility that Mn may disturb cellular development processes^49,50^ as well as the flow of genetic information that could influence protein synthesis^14–16^, including ER stress^21,40–43^.

Our global chemical-genetic sensitivity screen, followed by GO term enrichment of interaction network of participants, suggest that Mn disturbed anabolic metabolism pathways In line with this, we provide evidence to suggest that disruptions in the biosynthesis of amino acids through decreased expression of *ARG3* which is involved in the biosynthesis of arginine from ornithine carbamoyltransferase^51^. Previous works have suggested that ornithine deficiency causes hyperammonemia and neurotoxicity in humans^52^. Specifically, alteration of arginine and proline metabolism has been associated with development of ALS^53^. The impairment of amino acid biosynthesis can directly disrupt translation efficiency^54^, a process that is energetically very costly^23,24^. Furthermore, these events appear to be associated with inactivation of MAPK pathways that can lead to translation repression^23,55^, antiapoptotic activities^56^ and/or cell cycle arrest^23,44,56^. Interestingly, we identified and inferred significant impairment of genes involved in the cell cycle and MAPK pathways (Figs 1, 2 and 4) such as *CDC20* and *CLB2*, which correlated with our qPCR results. Cell cycle disruption has been associated with AD, PD and ALS^57^. At the same time, aberrations from strictly controlled of MAPK signaling pathway have also been implicated in the development of different human diseases including AD, PD and ALS^56,58^.

According to the results discussed above, Mn-induced toxicity in yeast appears to be associated with essential pathways linked to protein metabolism. In the current study we inferred that Mn may induce ER stress (Fig. 2), which was demonstrated through qPCR analysis showing up-regulation of *OST6* and downregulation of *OST2* (Fig. 4). This is in agreement with previous *in vitro* and yeast studies that suggested that the ATPase activity of ER gene *SPF1* is compromised under exposure to Mn resulting in severe ER stress^43^. Other works have suggested that Mn-induced ER stress can be mediated through iron depletion, increased phosphorylation of the eukaryotic translation initiation factor 2α (phospho-eIF2α)^59^, activation of *PERK* and *IRE1* signaling pathways^41,42^ and ER tumefaction^60^. An RNA-Seq approach in *Caenorhabditis elegans* revealed that Mn induced both up and down-regulation of ER-related protein families (*FKB* and *ABU*) which are both implicated in ER stress^40^. ER stress can trigger a signaling reaction known as the unfolded protein response (*UPR*), which induces adaptive programs that improve protein folding. In certain neurodegenerative diseases such as AD, ALS, HD and PD, when the cell damage is irreversible, *UPR* can also activate apoptosis^61,62^.

Moreover, we found that Mn could potentially influence glycosylation through*OST2* and *OST6*^63,64^. Aminoglycoside antibiotics have been proposed to introduce errors in post-translational modifications such as glycosylation and protein misfolding that can lead to destabilized membranes and chronic stress^65^. Other studies suggest that alterations of *SLC39A8* links Mn deficiency to inherited glycosylation disorders, specifically impairment of Mn-dependent enzymes activity, most notably the Golgi enzyme β-1,4-galactosyltransferase, which is essential for biosynthesis of the carbohydrates in glycoproteins^63^. Moreover, Golgi glycosylation defects may also be the result of *Gdt1p/TMEM165* deficiencies that stem from Golgi Mn homeostasis defects^64^. Collectively, this evidence suggest that ER stress in yeast treated with Mn, may be associated with the impairment of N-glycan biosynthesis^66^, which could consequently lead to arrest the protein biosynthesis.

Additionally, ER stress can be exacerbated by the impairment of endosome-to-Golgi retrograde trafficking^67^. Since the retromer complex, comprised of vacuolar protein sorting, is essential to the bidirectional transport between the trans-Golgi network and endosomes. It is one of the key vesicular trafficking pathways in the cell^68^, particularly the transport of protein to endoplasmic reticulum^67,69,70^.

Vacuole protein sorting appears disrupted in the presence of Mn. *VPS5* mutants are hypersensitive to Mn (Fig. 1B), and the PPI network analysis implicatedprotein/genes with similar function such as *Vps35*, *Vps29*, *Vps17* and *PEP8* (Fig. 2). Interestingly, previous studies have reported that Mn is linked to yeast *VPS1*, *VPS53 and PEP8*^71^. Vesicle transport is considered to play an important role in yeast and mammalian models of ALS as well^72^. Furthermore, other studies have verified Mn down-regulated the expression of *SNAP-25* and up-regulated the expression of *VAMP-2*, which interacted with Synaptophysin^73^. Using the FM1-43 dye (N-(3-Triethylammoniumpropyl)-4-(4-(Dibutylamino) Styryl) Pyridinium Dibromide), an excellent reagent both for identifying actively firing neurons and for investigating the mechanisms of activity-dependent vesicle cycling^74^, various authors verified that FM1-43-labeled synaptic vesicles treated with Mn resulted in an initial increase followed by a decrease in the number of vesicles^73,75^. Other studies linked Mn neurotoxicity to the disruption of genes with transport functions including *SLC30A10*, *ATP13A2* and *ZnT10*^19,20^.

Alternatively, Gitler *et al*.^76^ identified that YPK9 overexpression significantly rescued the ability of proteins to leave the ER and traffic to the Golgi, which reduced the toxic effects of α–syn intracellular accumulation and Mn toxicity, suggesting a close connection between genetic and environmental causes of neurodegeneration. Ypk9 is a yeast orthologue of human PARK9/ATP13A2, whose expression in animal models of PD is capable of rescuing neurodegeneration^76^. At the same time, Golgi dysfunction can lead to the rapid repression of rRNA and ribosomal proteins^23^, affecting protein biosynthesis. Indeed, we observed that translation arrest in yeast was notably increased after 24 hours of exposure to Mn (decreasing of β-galactosidase activity), suggesting that long or chronic exposure of Mn^2+^ appears more effective in yeast than short or acute exposure which is similar to observations made by others^11,77^. We conducted the same analyses using other divalent cations such as Ca^2+^, Mg^2+^ and Zn^2+^ at equivalent or higher concentrations than used with Mn to determine if this was a general effect of metal stress and did not observe decreased rates of expression like the dose-dependent response to Mn seen in Fig. 3C. In addition, our ribosome profile analysis revealed a reduction in heavy polysomes fractions in response to Mn suggesting that Mn reduces efficiency of translation (Fig. 3B). This may be in agreement with a recent study in human SH-SY5Y cells that identified Mn-induced ER stress associated with increased phosphorylation of translation initiation factor *eIF2α*^59^. Similar profiles have been reported when using anti-translation drugs including pactamycin and harringtonine^78^.

Furthermore, we verified direct impairment of protein biosynthesis, including disruption of ribosome biogenesis due to down-regulation of the genes *NOP1*, *NSR1*; although this can be partially composed through up-regulation of the gene *RPS15*. A review at the CTD^48^ revealed that *RPS15* is a marker of Disease Progression, including memory impairment in transgenic mice modelingAD treated with copper^79^ as well as RPL14, potentially affected by Mn, is a marker of PD), which have been observed in case of residential exposure to maneb,. It is very interesting because, while paraquat is a derived of bipyridine, maneb is a polymeric complex of Mn. Unpublished studies from our group have identified maneb-induced impairment of protein biosynthesis in cerebellar granule neurons.

## Final Considerations

Literature in this field has consolidated robust hypotheses regarding Mn induced-neurotoxicity and neurodegeneration that include mitochondrial dysfunction, energy impairment, oxidative stress, disruption of neurotransmitters, ER stress, neuroinflammation, DNA damage and epigenetic alterations, apoptosis, autophagy, and many others^9,10,80,81^. However, occasionally, the accuracy of these hypotheses is challenged in different models. All processes cited above either occur after the process of protein synthesis is completed and/or are directly linked to it. In this study we identified protein synthesis as a key target of Mn-induced toxicity in *S*. *cerevisiae*. Defects in protein synthesis have been documented in different neurodegenerative disorders in humans. Since Mn-induced toxicity has been linked to human neurodegenerative disorders, the data presented in the current study may provide a connection between Mn-induced toxicity and human neurodegenerative disorders through the process of protein synthesis. Altogether, our findings provide strong evidence that Mn-toxicity can occur at multiple levels simultaneously, which appear to be associated with disruption of orchestrated essential pathways including metabolism and protein biosynthesis. In this way the presented study adds to our current understanding of the Mn-induced mode of toxicity. Additional experiments with mammalian models must be conducted to validate if these findings apply to other systems.

## Experimental

### Gene expression analysis

#### Manganese sensitivity/resistance screening using yeast gene deletion array

Approximately 4700 MATa haploid yeast, S. cerevisiae strains (BY4741, MATa ura3Δ0 leu2Δ0 his3Δ1 met15Δ0) from the non-essential Gene Deletion Array (yGDA) were manually arrayed onto agar plates as previously described by Alamgir, *et al*.^82^ in the presence or absence of sub-inhibitory concentration (a high concentration of a bioactive/toxic compound where growth of a WT strain is not completely inhibited) of MnCl_2_ (1.35 mM). Plates were incubated at 30 °C overnight. Finally, digital images of plates were used to analyze the growth of individual colonies, by automatized visual density comparison between control and their respective Mn treatment through of the available in-house and online software “SGAtools” from the University of Toronto^83^. The experiment was repeated between three and five times (Supplementary Material, Table SM 1). Colonies that showed 30% reduction or more in at least three repeats were considered hits^84–86^.

#### Transcriptomics experiment using q-PCR

The quantitative PCR (q-PCR) assay has been used to study the effects of a gene deletion on expression under specified conditionsgene deletion in previous works^87,88^. Primers of selected genes were synthetized (Table 2). Total RNA was isolated from each strain, using a Qiagen RNA isolation kit; followed by cDNA construction, using an iScript cDNA synthesis kit and finally used SYBR green supermix (Bio-Rad) for the multiplex real time PCR assay; according to the instructions of the manufacturer. The quantification of mRNA was performed by q-PCR on a Rotor-Gene RG-300 from Corbett research, according to Samanfar *et al*.^85^.

**Table 2:** Genes selected for q-PCR analysis. Primer sequences and associated parameters are included.

#	Gene Symbol	Sequence (5′->3′)		Template strand	Length	Start	Stop	Tm	GC (%)	Self complementarity	Self 3′ complementarity
1	OST2	**Forward primer**	TCCCGCTAAAAACGCATTGA	Plus	20	516602	516621	58.48	45	4	2
		**Reverse primer**	CAGCACGTCATCTGCAGTCT	Minus	20	516803	516784	60.39	55	6	3
		**Product length**	202								
2	OST6	**Forward primer**	CGCTGACAACTACCCACTGT	Plus	20	233594	233613	59.97	55	3	3
		**Reverse primer**	TGGCAACTCATGCCGTTACT	Minus	20	233692	233673	59.96	50	4	2
		**Product length**	99								
3	STT3	**Forward primer**	TTCGGTGACTTCGTGAAGGG	Plus	20	453244	453263	59.97	55	6	2
		**Reverse primer**	TCAAGGCAGAAAGTCCGACC	Minus	20	453352	453333	59.97	55	5	3
		**Product length**	109								
4	DSK2	**Forward primer**	GGACCCTAATGCCGGTATGG	Plus	20	819486	819505	59.96	60	6	3
		**Reverse primer**	TTCGTGTTGGAGCCTTCCTC	Minus	20	819578	819559	59.97	55	3	1
		**Product length**	93								
5	ARG3	**Forward primer**	GTTGCTGAGAGAAACGGTGC	Plus	20	269462	269481	59.76	55	5	2
		**Reverse primer**	GCTTGGCCTGTTTCGCAAAT	Minus	20	269588	269569	60.32	50	4	2
		**Product length**	127								
6	ICL1	**Forward primer**	ACCCAGCCTTTGGATGAAGG	Plus	20	285532	285551	59.96	55	4	2
		**Reverse primer**	GTTACAGAGGTGGGACGCAA	Minus	20	285764	285745	59.97	55	3	1
		**Product length**	233								
7	CLB2	**Forward primer**	CAGAGACAGACGGTGCATGT	Plus	20	772641	772660	60.04	55	4	3
		**Reverse primer**	CAGCTGCTGCACACAATGAG	Minus	20	772871	772852	60.11	55	7	3
		**Product length**	231								
8	CDC28	**Forward primer**	GCCAAGCTTTCCTCAATGGC	Plus	20	560815	560834	60.11	55	6	2
		**Reverse primer**	GGGTCATACGCGAGGAGTTT	Minus	20	560916	560897	59.82	55	4	1
		**Product length**	102								
9	rpl14A	**Forward primer**	TTGGCCGCTATCGTCGAAAT	Plus	20	431999	432018	60.18	50	6	3
		**Reverse primer**	TTGCCAGCACTTTTTCGTAGC	Minus	21	432120	432100	60	47.62	4	2
		**Product length**	122								
10	rpl14B	**Forward primer**	ACCTAAAACCCACCGTGGAC	Plus	20	104415	104434	59.89	55	7	3
		**Reverse primer**	GTTGGCGGTCCCTGAACATA	Minus	20	104492	104473	60.04	55	3	2
		**Product length**	78								
11	TIF1	**Forward primer**	ACTGGTAAGACCGGTACCTTTT	Plus	22	555194	555215	59.03	45.45	6	2
		**Reverse primer**	GCTTGAGGAGCCTTGACAGA	Minus	20	555264	555245	59.68	55	4	1
		**Product length**	71								
12	HYP2	**Forward primer**	TGAACATGGACGGTGACACT	Plus	20	85983	86002	59.24	50	5	3
		**Reverse primer**	GCTTCTTCACCCATAGCGGA	Minus	20	86112	86093	59.82	55	3	2
		**Product length**	130								
13	UFD1	**Forward primer**	GCGGCGGAAATGGTTTTGTA	Plus	20	589851	589870	59.76	50	3	2
		**Reverse primer**	ATTTTCCCGCCGAAGTTTGC	Minus	20	589968	589949	60.04	50	3	2
		**Product length**	118								
14	UFD2	**Forward primer**	CGGCGAAAGCAATCGTTCAA	Plus	20	121396	121415	60.11	50	4	3
		**Reverse primer**	GTGCCTCAAGGCTCAACTCT	Minus	20	121511	121492	59.96	55	6	1
		**Product length**	116								
15	CDC48	**Forward primer**	CCAGTACCAGGGGGACCATA	Plus	20	237886	237905	60.03	60	4	2
		**Reverse primer**	CAGTGAGGAAAGGCGACCAT	Minus	20	238201	238182	60.04	55	3	2
		**Product length**	316								
16	UBC11	**Forward primer**	ATCGTCTACGGGAAACGCAC	Plus	20	958728	958747	60.46	55	5	1
		**Reverse primer**	AGTAGAAGAGGGTGGTTGCG	Minus	20	958827	958808	59.39	55	2	2
		**Product length**	100								
17	CDC20	**Forward primer**	TTGCGTCCCCAACAAAGCTA	Plus	20	289873	289892	60.18	50	4	2
		**Reverse primer**	ATTAACGGTGGTGCCCCAAT	Minus	20	290059	290040	59.96	50	6	2
		**Product length**	187								
18	MCM2	**Forward primer**	GTGGCCAATCTTTCGTCTGC	Plus	20	175437	175456	59.83	55	6	2
		**Reverse primer**	CGCGCTGCTCAATTATTGCT	Minus	20	175602	175583	59.97	50	7	3
		**Product length**	166								
19	NSR1	**Forward primer**	CTAGAGCCTTCTTGGCGTCC	Plus	20	806681	806700	60.18	60	4	2
		**Reverse primer**	CCGTCCGTATCCCAACACAT	Minus	20	806773	806754	59.82	55	2	2
		**Product length**	93								
20	NOP1	**Forward primer**	ATTGCCCCAGGCAAGAAAGT	Plus	20	427853	427872	60.18	50	7	1
		**Reverse primer**	TCTCTGCCTGGTCTGTGAGA	Minus	20	427980	427961	59.89	55	4	3
		**Product length**	128								
21	URA2	**Forward primer**	CAAATTCTGGATGGCGCCTG	Plus	20	166665	166684	59.9	55	6	2
		**Reverse primer**	ACGGAAGAAGCAATCGCTGA	Minus	20	166793	166774	60.04	50	6	2
		**Product length**	129								
22	MCM2	**Forward primer**	GTGGCCAATCTTTCGTCTGC	Plus	20	175437	175456	59.83	55	6	2
		**Reverse primer**	CGCGCTGCTCAATTATTGCT	Minus	20	175602	175583	59.97	50	7	3
		**Product length**	166								

### Protein synthesis analysis

#### Total rRNA analysis

The yeast wild type strains were preincubated and grown overnight, then grown on YPD media at 30 °C to an OD_600_ of 0.8–1.0, in either the absence or presence of Mn (1.5 mM or 3 mM), for 0.75, 1 and 24 hours respectively. Total RNA was isolated from each strain using a Qiagen RNA isolation kit (RNeasy mini kit). RNA electrophoresis was carried out in 1X MOPS running buffer diluted from 10X MOPS buffer [40.8 g 3-(N-morpholino) propanesulfonic acid (MOPS); 6.8 g sodium acetate; 3.8 g ethylenediamine tetraacetic acid (EDTA)]. Volume was completed up to 1000 ml by the addition of ultrapure water treated with diethyl pyrocarbonate (DEPC), and the pH was adjusted to 7.0 using sodium hydroxide (NaOH)). The concentration of agarose used in the RNA gels was 1.2% (w/v). Samples were prepared in 80% v/v deionized formamide, heated at 65 °C for 5 minutes, then immediately cooled on ice. Before loading the samples on the gel, 1/10th of sample volume 10X RNA loading dye (0.0125 g Bromophenol Blue; 10 µl 0.5 M EDTA; 2.5 ml 100% glycerol; 2.5 ml DEPC-treated water; mixed by vortexing and autoclaved) was added to the samples for a final concentration of 1 × (1 µg).

#### β-Galactosidase expression assay

The efficiency of translation was quantified using an inducible β-galactosidase reporter gene in the p416 plasmid^82,89^. β-galactosidase is a model of intracellular protein synthesis that can provide a profile of aberrancy in the rate of protein synthesis and an estimate of gene expression. Thus, yeast cells transformed with p416 plasmid were preincubated for 1 hour and then exposed to a crescent toxicological curve of Mn (0.25–3 mM) and other divalent ions (Ca^2+^, Mg^2+^ and Zn^2+^ at 0.5, 1.5, 5 mM and a higher 15 mM concentration for Mg^2+^) for 3 hours at 30 °C, followed of spectrophotometric determination of β-galactosidase activity^82^. Metal ion concentrations were suggested by previous works^90–93^.

#### Ribosome profile analysis

Ribosome profiling^94^, allows for the monitoring of translation dynamics *in vivo*. Yeast wild type strains were preincubated 1 hr and then grown on YPD media at 30 °C to an OD_600_ of 0.8–1.0, in the absence or presence of 3 mM Mn, for 1 hr and 24 hrs respectively at 30 °C. Immediately before harvest, cycloheximide was added to all samples, to a final concentration of 100 μg/ml, and the culture was incubated again at 30 °C for 15 minutes, followed by a cold snap in an ice water bath. Cells were harvested, washed with a cycloheximide/water solution (100 μg/ml) and centrifuged at 4000 rpm for 4 min at 4 °C using a Sorvall SLA-1500 rotor to separate the supernatant. Cell pellets were resuspended in 10 ml of ice-cold lysis buffer A (YA buffer: 10 mM Tris-HCl [pH 7.4], 100 mM NaCl, 30 mM MgCl_2_, cycloheximide 50 μg/ml, heparin 200 μg/ml) and centrifuged at 4000 rpm for 4 min at 4 °C (Sorvall SS34 rotor) twice. Pellets were resuspended in 750 µl of YA buffer, lysed by vortexing with glass beads, transferred to microtubes, and centrifuged at 13000 rpm for 10 minutes at 4 °C. The supernatant was preserved for the quantitative determination of total RNA, followed by fractionation on 10–50% sucrose gradients containing 50 mM Tris-acetate [pH 7.0], 50 mM NH_4_Cl, 12 mM MgCl_2_, and 1 mM dithiothreitol. The extract was centrifuged for 2 h at 40,000 rpm using a SW40-Ti rotor in a Beckman LE-80 K at 4 °C. The polysome profiles were analyzed via a Biocomp gradient station and the absorbance was recorded at 254 nm using a spectrophotometer (Bio-Rad Econo UV monitor) coupled with the Biocomp station. In this method, free mRNAs from the top fractions were separated from polysome-associated mRNAs from the bottom fractions^95^.

### Protein-protein interaction (PPI) prediction and gene ontology (GO) analysis

A PPI network can be described as a heterogeneous network of proteins joined by interactions as edges. Protein network and GO enrichment analysis were based on the data from the current project and analyzed using the STRING database (http://string-db.org)^38^. Additional GO analysis was conducted at the Comparative Toxicogenomic Database – CTD (http://ctdbase.org/)^48^ to test the hypothesis of a conserved mode of action of Mn between yeast and humans. Both STRING and the CTD database were accessed on November 18^th^, 2018.

### Data analysis

The results were expressed as mean ± sem of at least three independent experiments. To detect statistically significant differences, ANOVA (analysis of variance) followed by Bonferroni’s tests was be used; preceded of single t-test analysis between pairs of treatments. Fitting and statistical analyses were performed using GraphPad Prism (GraphPad 4.0 Software Inc, San Diego, CA, USA).

## Supplementary information

SUPPLEMENTARY MATERIAL

## Data Availability

All data generated and/or analyzed during this study are included in this published article and/or its Supplementary Material Files).
