# Supplementary material for: Manganese-induced cellular disturbance in the baker’s yeast, Saccharomyces cerevisiae with putative implications in neuronal dysfunction
Source: Sci Rep. 2019 Apr 25;9:6563. doi: 10.1038/s41598-019-42907-2 (PMC6484083; doi:10.1038/s41598-019-42907-2)
Supplement: Supplementary file 1 — SUPPLEMENTARY MATERIAL [file 41598_2019_42907_MOESM1_ESM.pdf]

Manganese-induced cellular disturbance in the baker's yeast, *Saccharomyces cerevisiae* with putative implications in neuronal dysfunction

Raúl Bonne Hernández[1,2*+], Houman Moteshareie[2+], Daniel Burnside[2], Bruce McKay[2] and Ashkan Golshani[2*+]

[1]Univ. Federal de São Paulo Departamento de Química. Laboratorio de Bioinorgânica e Toxicologia Ambiental – LABITA. Rua Prof. Artur Riedel, 275, CEP: 09972-270. Diadema-SP, Brazil.

[2]Department of Biology. Carleton University. 209 Nesbitt Biology Building. 1125 Colonel by Drive. Ottawa, ON, K1S 5B6.

*Corresponding author: rbhernandez@unifesp.br, ashkan_golshani@carleton.ca

+These authors contributed equally to this work

SUPPLEMENTARY MATERIAL - SM

Table – SM 1: Full list of genes significatively affected, after exposure for Mn^{2+}

	ORF	Gene	ave (control)	sd	n	ave (MnCl2)	sd	n	Score (Control-MnCl2)	Sensitivity Intensity (%)
1	YGL168W	HUR1	1.170865	0.036075	3	0.002035	0.002035	3	1.16883	117
2	YGR081C	SLX9	1.152335	0.011215	3	0.197255	0.063595	3	0.95508	98
3	YDR305C	HNT2	1.083995	0.091093	4	0.33684	0.185545	4	0.747155	77
4	YOR069W	VPS5	1.208313	0.174882	4	0.4927775	0.173317	4	0.7155355	80
5	YPR119W	CLB2	1.097477	0.052092	3	0.4377133	0.091313	3	0.6597637	70
6	YFR039C	OSW7	1.08166	0.22522	3	0.425725	0.198735	3	0.655935	69
7	YJL088W	ARG3	1.053865	0.017035	3	0.430625	0.016145	3	0.62324	65
8	YOR035C	SHE4	0.90118	0.051217	4	0.3129625	0.047859	4	0.5882175	55
9	YPR046W	MCM16	0.9537067	0.012492	3	0.3797467	0.076557	3	0.57396	56
10	YOL113W	SKM1	1.244925	0.138617	4	0.707675	0.132938	4	0.53725	68
11	YBR164C	ARL1	1.03298	0.017411	4	0.5271025	0.055719	4	0.5058775	52
12	YOR298C-A	MBF1	1.157615	0.040069	4	0.66817	0.099346	4	0.489445	58
13	YPR155C	NCA2	0.99624	0.019924	3	0.5248333	0.042673	3	0.4714067	47
14	YKR023W	YKR023W	1.14421	0.105178	4	0.6748275	0.112328	4	0.4693825	55
15	YJL083W	TAX4	1.09023	0.06004	3	0.66728	0.07136	3	0.42295	48
16	YDL190C	UFD2	1.028383	0.047172	4	0.63136	0.06979	4	0.397023	41
17	YLR420W	URA4	1.458403	0.010124	3	1.067497	0.039561	3	0.390906	73
18	YGR053C	YGR053C	1.075375	0.011815	3	0.696855	0.015395	3	0.37852	43
19	YGR236C	SPG1	1.149022	0.082789	4	0.7781675	0.085497	4	0.3708545	47
20	YHR105W	YPT35	1.09611	0.026874	4	0.7262625	0.043143	4	0.3698475	43
21	YMR209C	YMR209C	1.03769	0.03663	3	0.678735	0.033595	3	0.358955	38
22	YJL160C	PIR5	1.03089	0.00348	3	0.67749	0.02608	3	0.3534	37
23	YOR043W	WHI2	0.982135	0.05962	4	0.635275	0.096336	4	0.34686	34
24	YML019W	OST6	1.25041	0.093263	3	0.918	0.074999	3	0.33241	52
25	YOR356W	CIR2	1.09134	0.058099	4	0.7641175	0.097666	4	0.3272225	39
26	YPL259C	APM1	1.088425	0.091109	4	0.7641075	0.116263	4	0.3243175	39
27	YOR196C	LIP5	1.060465	0.043026	4	0.7422575	0.075072	4	0.3182075	36
28	YDR503C	LPP1	1.02099	0.021008	5	0.706006	0.054774	5	0.314984	33
29	YBL031W	SHE1	0.90021	0.061126	4	0.5860325	0.045859	4	0.3141775	25
30	YOR339C	UBC11	1.140852	0.096454	4	0.82858	0.045355	4	0.312272	41
31	YAR002C-A	ERP1	1.075498	0.051323	4	0.76688	0.094286	4	0.308618	36
32	YHR146W	CRP1	1.097638	0.043653	4	0.79051	0.042915	4	0.307128	38
33	YHR130C	YHR130C	1.072762	0.078784	4	0.765925	0.078998	4	0.306837	36
34	YKL092C	BUD2	1.0376	0.100048	4	0.73092	0.019977	4	0.30668	33
35	YDR202C	RAV2	1.028592	0.06063	4	0.7263525	0.0449	4	0.3022395	32
36	YEL039C	CYC7	1.043434	0.034105	5	0.748896	0.068135	5	0.294538	33
37	YBR034C	HMT1	1.032915	0.040322	4	0.7421125	0.120755	4	0.2908025	31
38	YDR506C	GMC1	1.032612	0.034063	5	0.751172	0.082956	5	0.28144	31
39	YBL039C	URA7	0.9790025	0.042451	4	0.700915	0.062075	4	0.2780875	26
40	YEL038W	UTR4	0.993002	0.044155	5	0.722974	0.079207	5	0.270028	26
41	YJR147W	HMS2	1.099748	0.021829	4	0.8342575	0.081935	4	0.2654905	34
42	YBR214W	SDS24	1.049032	0.063572	4	0.7838125	0.077667	4	0.2652195	30
43	YML062C	MFT1	1.362	0.034338	3	1.098283	0.045214	3	0.263717	56
44	YFL011W	HXT10	1.056252	0.027572	5	0.805832	0.080767	5	0.25042	29
45	YNL068C	FKH2	0.9983325	0.050421	4	0.75155	0.03427	4	0.2467825	25
46	YPL116W	HOS3	1.164265	0.053946	4	0.9195375	0.032581	4	0.2447275	37
47	YBR200W	BEM1	0.7616675	0.076916	4	0.5195475	0.084815	4	0.24212	8
48	YBR036C	CSG2	1.058998	0.031558	4	0.82043	0.02785	4	0.238568	28
49	YBR073W	RDH54	1.033402	0.044144	4	0.8039275	0.04389	4	0.2294745	26
50	YER065C	ICL1	1.11932	0.064358	5	0.891106	0.050358	5	0.228214	32
51	YER056C	FCY2	1.07875	0.035131	5	0.858666	0.050824	5	0.220084	28
52	YER067C-A	YER067C-A	1.054336	0.036081	5	0.843586	0.059283	5	0.21075	25
53	YDR431W	YDR431W	1.140604	0.054012	5	0.930166	0.021056	5	0.210438	33
54	YDR216W	ADR1	1.099082	0.027995	4	0.8904775	0.071701	4	0.2086045	29
55	YOR120W	GCY1	1.01461	0.023321	4	0.818365	0.062186	4	0.196245	21
56	YDR445C	YDR445C	1.001802	0.026957	5	0.81015	0.062413	5	0.191652	19
57	YHR104W	GRE3	1.029137	0.017892	4	0.8382225	0.049999	4	0.1909145	21
58	YBR129C	OPY1	1.034863	0.038183	4	0.852305	0.015116	4	0.182558	21
59	YAL020C	ATS1	0.9270175	0.039382	4	0.7445325	0.027469	4	0.182485	12
60	YDR097C	MSH6	1.025058	0.039329	4	0.843625	0.015504	4	0.181433	20
61	YML009c	MRPL39	1.058793	0.051166	3	0.88467	0.019255	3	0.174123	22
62	YHL031C	GOS1	0.960635	0.045899	4	0.7893075	0.036217	4	0.1713275	14
63	YOL155C	HPF1	1.013653	0.110044	4	0.844445	0.09764	4	0.169208	18
64	YBL085W	BOI1	1.155473	0.077384	4	0.9927825	0.095165	4	0.1626905	30
65	YBR050C	REG2	1.041288	0.043482	4	0.915575	0.061218	4	0.125713	16
66	YOR347C	PYK2	0.9492275	0.029227	4	0.8269225	0.036802	4	0.122305	8
67	YER151C	UBP3	0.951364	0.01368	5	0.830952	0.027866	5	0.120412	8
68	YBL095W	MRX3	1.018275	0.04173	4	0.9388825	0.034991	4	0.0793925	10

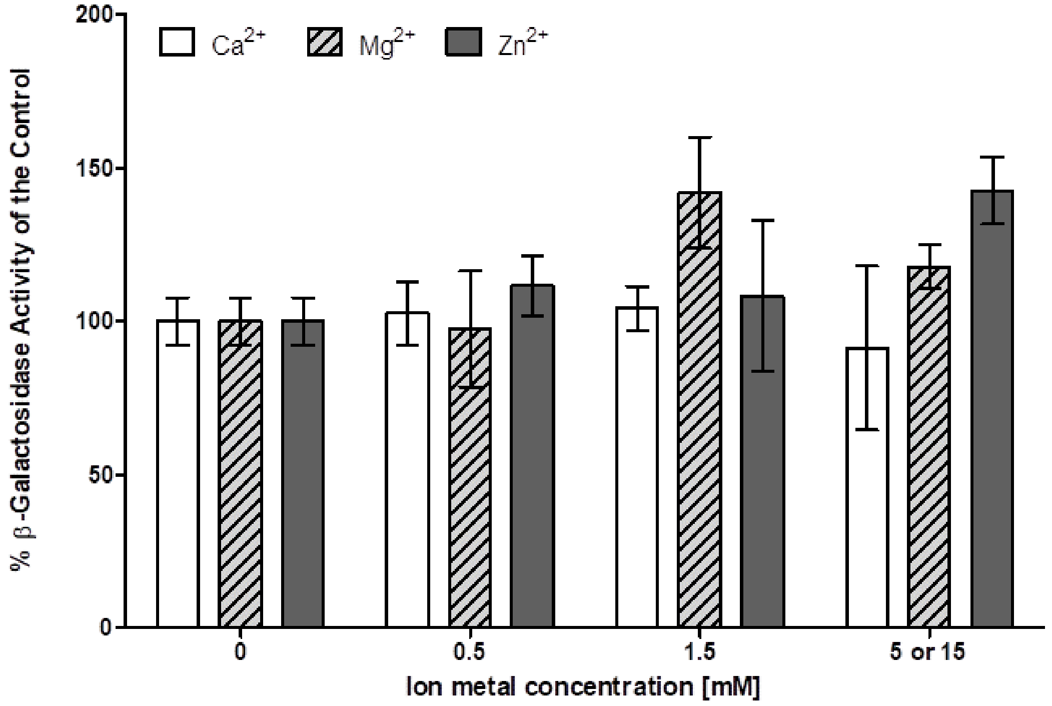

Figure – SM 2: The relative expression of β-galactosidase in response to the presence of increasing concentrations (0 – 5 mM) of Ca^{2+}, Mg^{2+} and Zn^{2+} ions. In the specific case of the Mg^{2+}, the highest concentration was 15 mM.

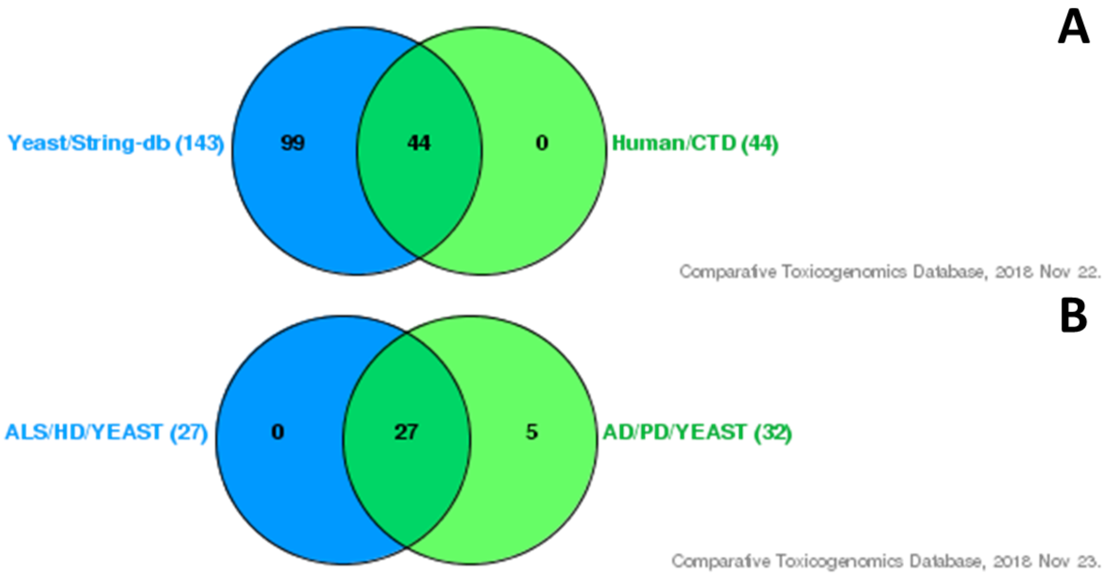

Figure – SM 3: Humans and yeast share orthologous genes potentially linked to neurodegenerative disorders. Approximately 31% (44 proteins/genes) of the inferred network for hits (143 proteins/genes) affected by manganese have homologues in human (**A**), of which approximately 73% (32 proteins/genes of the 44 homologues in human) are potentially associated with neurodegeneration (**B**).

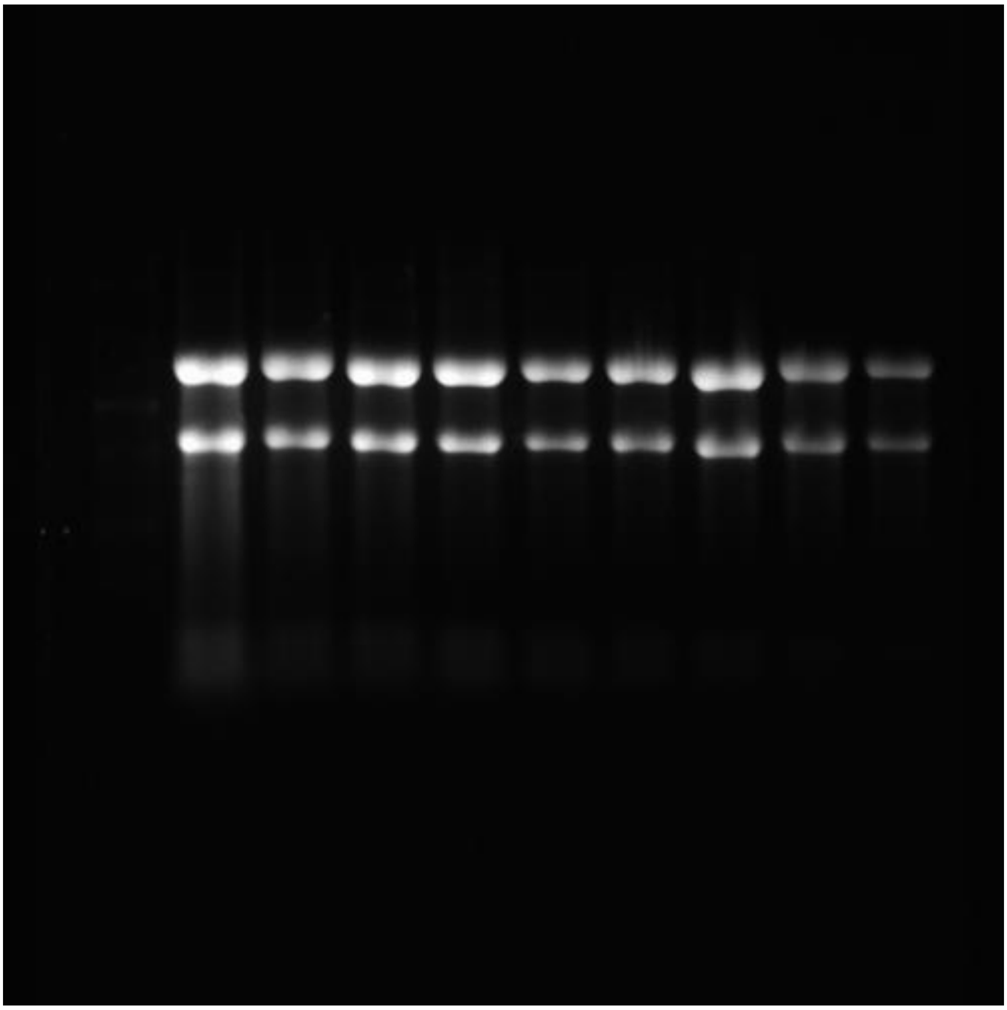

Figure SM 4: Unedited photo of the RNA gel presented in Figure 3a.
